# Associations between dental care approachability and dental attendance among women pregnant with an Indigenous child: a cross-sectional study

**DOI:** 10.1186/s12903-021-01816-5

**Published:** 2021-09-17

**Authors:** Yuan Gao, Xiangqun Ju, Lisa Jamieson

**Affiliations:** 1 School of Public Health, The University of Adelaide, Adelaide, Australia; 2 Australian Research Centre for Population Oral Health, Adelaide Dental School, The University of Adelaide, Adelaide, Australia

**Keywords:** Aboriginal study, Access to healthcare, Approachability, Accessibility, Oral health

## Abstract

**Background:**

Oral health during pregnancy is vital for both mother and child. Indigenous Australians face many barriers in accessing dental care. Service approachability is one of the key domains in accessing health services. There is little empirical evidence of the association between service approachability and dental care attendance or oral health outcome. The aim of this study is to examine the relationship between dental service approachability on dental care attendance and self-reported gum disease among South Australian women pregnant with an Aboriginal child.

**Methods:**

Four hundred and twenty-seven women pregnant with an Aboriginal child completed questionnaires in both metropolitan and regional health settings in South Australia in 2011. Four variables related to approachability of dental services: (1) perception of need; (2) service-related health literacy; (3) oral health beliefs and; (4) trust and expectation of dental service. The association between service approachability-related factors, dental utilisation and self-reported gum disease during pregnancy were assessed using Generalised Poisson regression models, after adjusting for age, remoteness, employment status and education. Estimates were presented as adjusted prevalence ratios (APR).

**Results:**

Most participants (85.8%) reported a need for dental care, had positive oral health beliefs (88.3%) and had expectations towards dental care (86.2%). Dental service utilisation during pregnancy was low (35.7%). Many participants (78.0%) expressed knowing what to do if they needed dental care, while most (39.8%) doubted that dental care would be available the next day. Poor health service literacy was identified as a risk factor for non-optimal dental attendance (APR = 0.86, 95%CI 0.74–0.99). Perceived need for dental care was positively associated with self-reported gum disease (APR = 1.24, 95%CI 1.06–1.45).

**Conclusion:**

Inability to navigate the dental care system was a risk factor for poor dental attendance among South Australian women pregnant with an Aboriginal child. Perceived need for dental care was associated with gum disease.

**Supplementary Information:**

The online version contains supplementary material available at 10.1186/s12903-021-01816-5.

## Background

Indigenous Australians are those who identify as Aboriginal and/or Torres Strait Lander [1]. Indigenous Australians are the first residents of Australia, and have unique traditions, cultures, and languages [1]. However, Indigenous Australians have poorer oral health, and experience more oral health conditions compared with non-Indigenous Australians [2]. In the National survey of Adult Oral Health, Indigenous adults had significantly higher levels of untreated caries and missing teeth, and a lower prevalence of filled teeth, compared with non-Indigenous Australian [3].

Pregnant women are more affected by oral conditions due to hormonal and immunologic changes during pregnancy [4, 5]. Oral conditions during pregnancy may have adverse effects on both maternal and child health outcomes. Approximately 30 ~ 47% [6, 7] of pregnant women have experienced gingivitis during pregnancy, which leads to pain, uncontrollable bleeding, and difficulties in eating [8]. Periodontal disease, which stems from gingivitis, may increase risk of adverse maternal outcomes, such as systemic inflammation [9, 10] and preeclampsia [11, 12]. Maternal experience of dental caries during pregnancy is a contributing factor of early childhood caries (ECC) among children [13]. ECC affects children’s eating, speech and self-confidence [13]. Experience of dental disease in childhood increases the risk of experiencing dental disease in later life [14, 15].

To maintain good oral health, annual dental check-ups are essential [16]. A higher proportion of non-Indigenous Australians attend a dentist once or more a year (60.3%) [17] compared with Indigenous Australians (15–38%) [18, 19]. The low utilisation of dental care among Indigenous Australians may arise from a range of barriers Indigenous Australians face in regard to accessing timely, culturally appropriate and affordable dental care. Specifically, factors affecting dental care uptake of Indigenous Australians include cultural appropriateness of service [20, 21], remoteness of residency [22], cost [23] and experience of discrimination in previous receipt of health services [24].

This study is based on the theory developed by Levesque and colleagues [25] in accessing health service (see Additional file 1: Figure S1). We were especially interested in one of the domains, which is the effect of service approachability on utilisation of dental care and oral health outcomes. The service approachability is corresponding to one’s ability of how to perceive the demand. [25]. Levesque and colleagues [25] noted that approachability of a health service should enable people who need the service to identify that the service exists, can be reached, and will have an impact on their health [25]. On the demand side [25], service approachability is related to one’s ability to perceive the need of a service, which is constructed by one’s health literacy, health belief and expectation and trust of the service. Individual health literacy is related to one’s ability to access, understand and apply health information [26]. Health literacy was referred to service-related health literacy, including knowledge of system navigation, which is essential because it is the first step in interacting with the heath care environment [27]. In the context of oral health, a belief in good oral health is important to ensure dental services are utilised; such beliefs in oral health can lead to behaviour changes, for example, leading one to seek health care in the first instance. Meanwhile, parental oral health beliefs also have impacts on offspring and can predict the uptake of dental care as children grow older [28]. Finally, trust and expectation of the health service play indispensable roles in accessing health care, especially in the Indigenous Australian context. Due to long lasting legacies of colonial practises and laws, including cultural discrimination, lack of trust is one of the primary causes of poor uptake of health services among Indigenous Australians [29].

Other researchers have, in recent years, applied the model developed by Levesque [25] when working with marginalised populations, such as refugees [30] and Indigenous people [27]. However, all prior research used the model to structure reviews, not to examine the inherent associations of each of the domains with a given service utilisation and its health outcome. The aim of this study was innovative in applying the Levesque model to examine the relationship between dental service approachability on the demand side with dental care attendance and self-reported gum disease among women pregnant with an Aboriginal child in South Australia. The hypothesis was that participants with a perceived need for dental care would have a higher uptake of dental care, resulting in better oral health outcome.

## Methods

### Study design

This study is a cross-sectional study; and data for the study were collected during 2011–2012 as part of the baseline data collection of an early childhood caries intervention among Indigenous children in South Australia [31, 32].

### Setting and recruitment

Participants were recruited through the antenatal clinic of hospitals and Aboriginal Community Controlled Health Organisations in South Australia in both metropolitan and regional locations. During data collection, researchers and staff members in health settings would approach potential participants and to provide information about the study, before obtaining written, informed consent. Convenience sampling was adopted, and criteria were: (1) Participants must be the pregnant residents of South Australia, and (2) were expecting an Aboriginal Australian baby or babies. The questionnaire included items used in the Australia national dental survey [33], and had been pilot tested and discussed by members in Indigenous communities and Aboriginal Maternal Infant Care workers. There were 23 domains with a wide range of oral health information in the questionnaire, including dental health, dental behaviours, dental cost, dental perceptions, oral health belief, etc. Items used in the study were oral health outcome, outcome of service utilisation, and factors related to dental care approachability [31, 32]. Recruitment commenced February 1, 2011 and ended on May 30, 2012. Participants who did not answer all questions were excluded from the study.

### Ethics and consent

Ethical approval was received from the University of Adelaide Human Research Ethics Committee, the Aboriginal Health Council of South Australia, the Government of South Australia, the Human Research Ethics Committee of Child, Youth and Women’s Health Service, and the Human Research Ethics Committees of participating Adelaide hospitals. The study was guided by an Indigenous reference group, World Health Organisation guidelines on ethical conduct in health research on Indigenous people [34], and local Indigenous South Australia principles. The study additionally used the Ethical Conduct in Aboriginal and Torres Strait Islander Health Research guidelines to obtain consent [35]. Parents of the participants provided signed informed consent for those being under the age of 16 years. Participants received a $50 voucher for reimbursement of time after completing the questionnaires.

### Development of service-oriented model of accessing dental care

Levesque and colleagues [25] developed a model that summarized the key determinants in accessing health service through a multi-level perspective (see supplementary Figure S1). The five dimensions that may be used to evaluate accessibility of a given health service, and include service: (1) approachability; (2) acceptability; (3) availability and accommodation; (4) affordability and; (5) appropriateness. The five dimensions reflect linear stages of a patient’s journey from the initial perception of requiring health care to the final accomplishment of receiving the required treatment. These five dimensions simultaneously correspond with five abilities for consumers: (1) ability to perceive; (2) ability to seek; (3) ability to reach; (4) ability to pay and; (5) ability to engage [25]. Factors that impacted service approachability (ability to perceive) were health literacy, health belief and expectation and trust of the service.

**Fig. 1:**
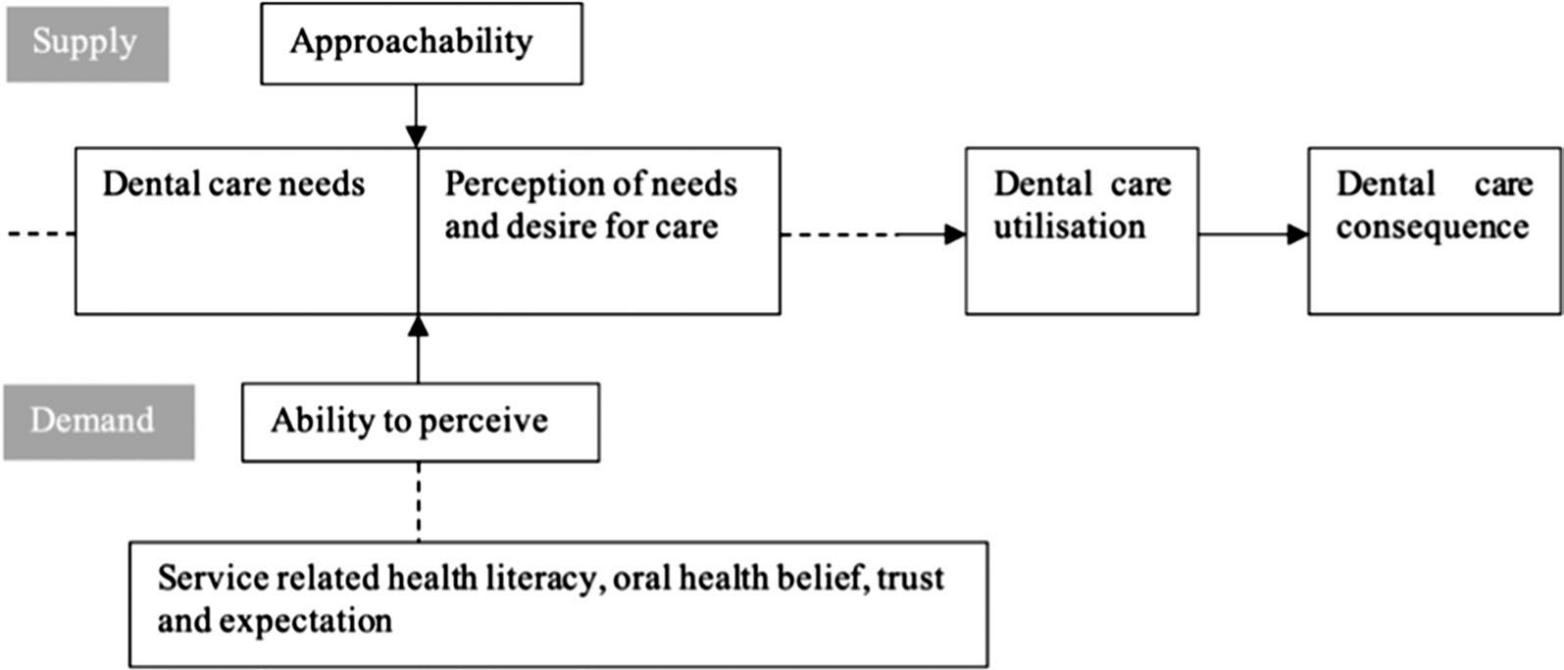
Service-oriented model of accessing dental care for women pregnant with an Indigenous child

To better fit the oral health context, we modified the model developed by Levesque (Fig. 1) [25]. Each factor was replaced by oral health-related, and dental service-oriented determinates. These included oral health service-related health literacy, which included literacy about dental system navigation, oral health beliefs of visiting a dentist, trust and expectations of a dental service, and perceived need for dental care. According to the modified model, different stages were linear from the perception of needing care to the accomplishment of the dental patient journey.

### Variables

According to the modified model, there were three factors impacting ability to perceive: dental service health literacy, oral heath beliefs, and trust and expectations of the dental provider. With the addition of perceived need for dental care, there were thus four dimensions measured in this study (see Additional file 1: Table S1 and Figure S2).

Dental service-related health literacy was measured by patient’s ability to navigate to the dental health system. Dental service-related health literacy was measured by “If you needed to visit the dentist tomorrow, would you know what to do?” and “Do you think there would be a dentist able to see you tomorrow?” (response options ‘yes’ or ‘no’). Dental health belief was measured by the question: “How important do you rate the following in relation to teeth?”, with ‘visiting the dentist’ being the domain of interest. Response options included: ‘extremely important’, ‘fairly important’, ‘doesn’t matter much’, ‘not very important’ and ‘not at all important’. To facilitate analysis, responses to this question were dichotomised into ‘extremely/fairly important’ and ‘doesn’t matter much/not very/not at all important’. Trust and expectation toward dental care was measured by the question “I believe going to the dentist would help my teeth”, and responses were re-dichotomised as ‘strongly agree’ and ‘not strongly agree/somewhat agree or doesn’t matter much’ from five sequential responds. The oral health outcome was measured by self-reported gum disease during pregnancy. The dental care utilisation outcome was measured by time of the last dental visit (≤ 1 year or 12 + months). Participant’s perception of need was measured by asking: “Do you think you need to see a dentist?” (response options ‘yes’ or ‘no’).

Social-demographic variables included age, employment status, education level and geographic remoteness of residential location. The definition of the remoteness of the resident location followed the Accessibility/Remoteness Index of Australia (ARIA+) [36], with the location subsequently categorised as “remote” and “non-remote area”. Age was presented as mean values in years plus standard deviation (SD) and was re-categorised as ‘34 years or less’ and ‘over 34 years’ to facilitate multivariable analysis. Education was categorised as ‘no schooling’, ‘primary/secondary education’, and ‘tertiary education’. Employment status was categorised as ‘employed’ or ‘receiving Centrelink payment/other’.

### Statistical analysis

Age was presented as means and standard deviations. All other variables were categorical, and thus presented as frequency and percentage. Chi-square tests were used in bivariate analysis, while adjusted prevalence ratios and their corresponding 95% confidence intervals were used in multivariable analysis using Generalised Poisson regression models [37] because the distribution of outcome variables was under-dispersed [38]. Factors related to service approachability (perceived need for dental care, oral health belief, dental service health literacy and trust and expectation toward service) were tested in bivariate analysis, with variables with statistically significant differences (*P* < 0.05) entered into multivariable models. Confounders were adjusted for, and included remoteness of residency, education level and employment status. Age was an additional confounder in the model involving dental attendance (Table 2), because studies have found pregnant women aged above 35 years old were more likely to access dental care [39], and we assumed that such women would be more experienced in health care seeking. Additional analyses were performed to examine the association between dental attendance and self-reported gum disease. Annual dental visit was entered into the regression model as an exposure for self-reported gum disease, and adjusted for remoteness, education and employment status. Variables with *P* < 0.05 in 2-sided α level were considered as being statistically significantly different in all analyses. Data were analysed using R version 3.6.1.

## Results

A total of 554 eligible participants were invited to take part in the study, with 427 (77%) providing consent and completing the questionnaire. The average age of participants was 25.3 $\pm \hspace{0.17em}$5.8 years (Table 1). Most participants reported having received primary/secondary education (70.3%), and approximately one-third (28.1%) had received tertiary education. Approximately 15% of participants were in current employment. The majority of participants lived in non-remote locations (86.9%).

**Table 1:** Sample demographic characteristics and service approachability-related factors among women pregnant with an Indigenous child in South Australia

	n	% (95% CI)
Total	427	100.0
*Age (mean, SD)*		25.28 ± 5.84
≥ ﻿35﻿ years	24	5.9 (3.8–8.6)
< 35 years	381	94.1 (91.3–96.1)
*Education level*		
No schooling	7	1.7 (0.6–3.4)
Primary/secondary education	298	70.3 (65.7–74.6)
Tertiary education	119	28.1 (23.8–32.6)
*Employment*		
Job	61	14.5 (11.3–18.2)
Other/centrelink payment	360	85.5 (81.8–88.7)
*Location*		
Non-remote	359	86.9 (83.2–90.0)
Remote	54	13.1 (1.0–16.7)
*Oral health outcome*		
Do you have gum disease/bleeding gums?		
Yes	182	42.7 (38.0–47.6)
No	244	57.3 (52.4–62.0)
*Dental service utilisation*		
Have you seen the dentist before?		
Yes	411	96.9 (94.8–98.4)
No	13	3.1 (1.6–5.2)
When did you last see a dentist?		
< 1 year	147	35.7 (31.0–40.5)
≥ 1 year (s)	265	64.3 (59.4–69.0)
*Perceiving need of dental service*		
Do you think you need to see a dentist?		
Yes	362	85.8 (82.0–89.0)
No	60	14.2 (11.0–17.9)
*Service-related oral health belief*		
How important do you rate the visiting dentist in relation to health?		
Extremely/fairly important	376	88.3 (84.8–91.1)
Doesn’t matter much/Not important	50	11.7 (8.8–15.2)
*Service-related health literacy (system navigation)*		
If you needed to visit the dentist tomorrow, do you know what to do?		
Yes	333	78.0 (73.8–81.8)
No	94	22.0 (18.2–26.3)
Do you think there would be a dentist able to see you tomorrow?		
Yes	168	39.8 (35.1–44.7)
No	254	60.2 (55.3–64.9)
*Trust and expectation*		
I believe going to dentist would help my teeth		
Strongly agree	367	86.2 (82.5–89.3)
Somewhat agree or don’t know	59	13.8 (10.1–17.5)

As shown in Table 1, 42.7% of participants reported having experience of gum disease. Almost all participants (96.9%) reported having seen a dentist in their lifetime. 85.8% of participants perceived a need for dental care. Of these, more than one-third (35.7%) had visited a dentist in the previous 12 months. Most participants (88.3%) perceived visiting a dentist to be very important. Of these, 36.7% of participants had attended for dental care in the last 12 months (Table 2). Approximately one quarter of participants (22.0%) reported not knowing what to do if they needed to visit the dentist the next day. Just over 60% (60.2%) of participants reported that they did not think a dentist would be able to see them the next day. Most participants (86.2%) strongly agreed that going to the dentist would help their teeth.

**Table 2:** Percentage, prevalence ratio (PR) and 95% CI of visiting dentist less than 1 year among women pregnant with an Indigenous child in South Australia (n = 427)

	% (95% CI)	PR 95% CI^a^	APR 95% CI^b^
*Perceiving need of dental service*			
Do you think you need to see a dentist?			
No	46.7(28.1–38.0)	Ref	–
Yes	32.9 (33.7–60.0)	1.24 (0.96–1.58)	–
*Service-related health literacy (system navigation)*			
If you needed to visit the dentist tomorrow, would you know what to do?			
Yes	39.9 (34.6–45.4)	Ref	Ref
No	14.9(8.4–23.7)	0.39(0.24–0.64)***	0.86(0.74–0.99)*
Do you think there would be a dentist able to see you tomorrow?			
Yes	44.0 (36.4–51.9)	Ref	Ref
No	28.3 (22.9–34.3)	0.64(0.49–0.82)***	0.91(0.81–1.02)
*Service-related oral health belief*			
How important do you rate the visiting dentist in relation to health?			
Extremely/fairly important	36.7 (31.8–41.8)	Ref	Ref
Doesn’t matter much/Not important	18.0 (8.6–31.4)	0.51(0.28–0.92)*	0.93(0.77–1.13)
*Trust and expectation*			
I believe going to dentist would help my teeth			
Strongly agree	37.1 (32.1–42.2)	Ref	Ref
Somewhat agree or don’t know	18.6 (9.7–30.9)	0.49(0.28–0.85)**	0.91(0.77–1.09)

PR, prevalence ratio; APR, adjust prevalence ratio

***p < 0.0001, **p < 0.001, *p < 0.05;

^a^Univariable analysis without any adjustment

^b^Reducing variable showing no statistical significance in univariable analysis and adjusting for remoteness, education level, employment and age

Table 2 shows the unadjusted and adjusted estimates from the multivariable analysis with visiting a dentist less than 12 months ago as the outcome and the service approachability factors as exposures. With the exception of perceived need for dental care, all factors related to service approachability were associated with dental service utilisation in the unadjusted analysis. After adjusting for remoteness of residency, education level, employment status and age, only one factor remained statistically significant: “not knowing what to do if needed to make a visit to the dentist the next day” (APR = 0.86, 95%CI 0.74–0.99).

Table 3 shows the analysis of service approachability factors with self-reported gum disease as the outcome variable. After adjusting for remoteness, employment status and education level, participants who perceived a need for dental care had 24% higher risk of having self-reported gum disease (APR = 1.24, 95%CI 1.06–1.45).

**Table 3:** Prevalence ratio (PR) and 95% CI of self-reported gum disease among women pregnant with an Indigenous child in South Australia (n = 427)

	% (95% CI)	PR 95% CI^a^	APR 95% CI^b^
*Perceiving need of dental service*			
Do you think you need to see a dentist?			
No	15.0 (7.1–26.6)	Ref	Ref
Yes	47.3 (42.0–52.5)	3.15(1.71–5.82) ***	1.24(1.06–1.45)**
*Service-related health literacy (system navigation)*			
If you needed to visit the dentist tomorrow, would you know what to do?			
Yes	41.1 (35.8–46.6)	Ref	–
No	47.9 (37.5–58.4)	1.18(0.92–1.51)	
Do you think there would be a dentist able to see you tomorrow?			
Yes	36.3 (29.0–44.1)	Ref	Ref
No	46.9 (40.6–53.2)	1.29(1.02–1.65)*	1.05 (0.94–1.17)
*Service-related oral health belief*			
How important do you rate the visiting dentist in relation to health?			
Extremely/fairly important	41.8 (36.7–46.9)	Ref	-
Doesn’t matter much/Not important	50.0 (35.5–64.5)	1.19(0.88–1.62)	
*Trust and expectation*			
I believe going to dentist would help my teeth			
Strongly agree	42.5 (37.4–47.7)	Ref	-
Somewhat agree or don’t know	44.1 (31.2–57.6)	1.03(0.76–1.41)	

PR, prevalence ratio; APR: adjust prevalence ratio

***p < 0.0001, **p < 0.001, *p < 0.05

^a^Univariable analysis without any adjustment

^b^Reducing variable showing no statistical significance in univariable analysis and adjusting for remoteness, education level, and employment

There were no statistically significant associations observed between dental attendance in the last 12 months and self-reported gum disease (Table 4).

**Table 4:** Prevalence ratio (PR) and 95% CI of self-reported gum disease and visiting dentist less than 1 year among women pregnant with an Indigenous child in South Australia (n = 427)

	% (95% CI)	PR 95% CI^a^	APR 95% CI^b^
*Visiting dentist* < *1 year*			
No	45.7 (39.6–51.9)	Ref	Ref
Yes	37.4 (29.6–45.8)	0.82(0.64–1.04)	0.95(0.86–1.05)

PR, prevalence ratio; APR, adjust prevalence ratio

***p < 0.0001, **p < 0.001, *p < 0.05

^a^Univariable analysis without any adjustment

^b^Reducing variable showing no statistical significance in univariable analysis and adjusting for remoteness, education level and employment

## Discussion

Our research sought to examine the relationship between dental service approachability, dental care attendance and self-reported gum disease among women pregnant with an Aboriginal child in South Australia using a modified version of the Levesque model. The findings showed that service-related factors were associated with dental attendance, which was consistent with the modified model. However, little effect was observed between service-related factors and self-reported gum disease, and no association was observed between dental attendance and self-rated gum disease. The results highlight the limitations of using the modified model in a quantitative study such as the one implemented.

Participants’ ability to navigate the dental care system was the key demand-side service approachability factor in utilising dental service. Previous research findings also reported Indigenous persons with higher skills in navigating dental services have higher compliance in long term dental treatment [40]. In this case, a person’s language capacity, knowing the information of location and contacts of dental clinics played an important role in the accomplishment of the dental care journey [40, 41]. However, due to the complexity of the Australian health system, many Indigenous and other socially or culturally marginalised groups struggle to adequately navigate the health system [42]. For instances, some public dental services are only available for children or young adults or government health care/concession card holders. For many states, Aboriginal people may need to contact local Aboriginal community-controlled health service first to access dental care [43]. For some Indigenous Australians, mainstream dental services (private or public) may be the only options for dental care, because dental services may not be provided by their local Aboriginal community-controlled health service. Barriers to successfully navigate mainstream dental services include language and cultural barriers. Empirical research has demonstrated that awareness of dental service availability may be limited for some Indigenous people [21], and also midwives [41]. Making dental service systems more navigable is crucial, given the negative impacts that poor dental care utilisation on oral health outcomes.

For Indigenous Australians to better navigate dental care systems, information in accessible formats is required [42]. According to Robards [42], navigation systems that integrate technologies, such as social media, may facilitate Indigenous Australians to better understand, connect and engage with dental care. Such interventions should be based in the Indigenous community setting. During the COVID-19 crises, Summer noted [44] that the application of social media channels shared through trustworthy local community networks enabled fast and effective health information sharing. Although dental care service provision may not always be available in the Aboriginal Community Controlled Health Organisation setting, such organisations had an indispensable role in the dissemination of health information, and a leading role of enhancing communication among Indigenous communities [44].

Based on these findings, future navigation programs that embrace social media and related technology might be more effective and economically friendly for women pregnant with an Indigenous child. Such services should be easy to contact to make health system navigation more approachable and understandable. Navigation support is just one example of improving system navigation. The health navigator program—targeting both Indigenous and non-Indigenous Australians—was increasingly used among patients with chronic disease who have difficulties in accessing health service, which improved the process of care [45]. There is evidence [46, 47] that Indigenous Liaison Officers can improve the engagement of Aboriginal families with health professionals, and may have a positive impact on diagnosis. There are some Aboriginal Liaison Programs for dental care [48, 49], although no study specifically examined its effect on uptake of dental care, the project was proven to be successful in dental referral to mainstream dental service [49]. There has been a Midwifery-Initiated Oral Health Dental Service program. In this program, midwives provided oral assessments and referrals to local and free public dental care for pregnant women. The referral letter included the contact details of a dentist, a checklist of date of visit, number of visits and treatment to better navigate participants to the service and to facilitate them to complete the course of recommended treatment [50]. The program was effective and promising in the improved uptake of dental care, and may be a beneficial pathway forward to implement among Indigenous populations [51].

One of our study hypotheses was that participants who had a perceived need for dental care would have better oral health than their counterparts with no perceived need; however, this did not prove to be the case (APR = 1.24, 95% CI 1.06–1.45). This suggests that the motivations or reason for participants’ perceived need for dental care were mixed and complicated. For example, the last visit for a dental appointment may have been for a check-up (a good oral health-related behaviour) and because of a problem. Thus, “uptake of dental care within one year” was found to be a weak indicator for oral health outcome. “Reason for that last visit” would have been a more reliable indicator for the phenomenon we were aiming to measure.

Our study made it possible to compare aboriginal to non-aboriginal pregnant women. A higher demand for dental care among Aboriginal women during pregnancy can be observed in this study (85.8%) compared with non-Aboriginal pregnant women in the United States (50.1%) [52]. The rate of dental visit < 12 months in this study (35.7%) was very close to a comparable study in New Zealand (37.7%) [19]. While it is still lower than non-Aboriginal pregnant women (45.6%) [53], and figures from high income countries were more in dental attendance, with approximately 70–92% pregnant women reported to have accessed dental care in the last 12 months [6, 54].

This was the first study to describe dental uptake and service approachability, and to test the association with self-reported gum disease among women pregnant with an Indigenous child in Australia. Most of studies [27] focus on provision of transport and reduction of cost to improve the accessibility of health care for Aboriginal people. Little empirical research has focused on the phases before actual interaction with the health care service, including participant motivation and capability to contact the service. This study reiterates the importance of system navigation in accessing dental care, which might also give more directions to improve accessibility of primary health care for Indigenous people. Indications for future research include: (1) Dental health literacy on how to navigate dental systems is important in the access outcome of dental care. Navigation support could be integrated with technologies, based on local community networks and collaborating with midwives. (2) The effect that approachability of a given service has on health outcomes (dental attendance). Motivations for visiting a dentist differ, and this has an impact on oral health outcomes. Previous uptake of dental care was not a good indicator of oral health. There is a need for better analytical approaches, and different measures of exposures and outcomes to better illustrate the impact that utilisation of dental care has on oral health outcomes.

The study limitations were that social desirability bias may have influenced participant responses and no clinical data was collected to ascertain objective measures of dental health. This study was cross-sectional in design implying that no assumptions of causality could be made.

## Conclusion

Although dental care was recognised as being important among our sample of women pregnant with an Indigenous child in South Australia, dental utilisation was low. Ability to successfully navigate the dental care system was associated with regular dental attendance. Perceived need for dental care was associated with self-reported gum disease. No association was observed between service-approachability-related factors and self-reported gum disease.

## Supplementary Information

**Additional file 1.Appendixes**. **Appendix A: Figure S1:** A conceptual framework of access to health care [25]. **Appendix B: Table S1:** Questionnaire of factors impacting on dental service approachability. **Appendix C: Figure S2:** Variables corresponding to service-oriented model of accessing dental care.

## Data Availability

Data cannot be shared publicly because of privacy issues of the participants. Data are available from the University of Adelaide Data Access (contact via Australian Research Centre for Population Oral Health: arcpoh@adelaide.edu.au) for researchers who meet the criteria for access to confidential data.

## Abbreviations

APR: Adjust prevalence ratio
ARIA+: The Accessibility/Remoteness Index of Australia
PR: Prevalence ratio
ECC: Early childhood caries
