# Supplementary material for: Associations between dental care approachability and dental attendance among women pregnant with an Indigenous child: a cross-sectional study
Source: BMC Oral Health. 2021 Sep 17;21:451. doi: 10.1186/s12903-021-01816-5 (PMC8446472; doi:10.1186/s12903-021-01816-5)
Supplement: Supplementary file 1 — Additional file 1.Appendixes. Appendix A: Figure S1: A conceptual framework of access to health care [25]. Appendix B: Table S1: Questionnaire of factors impacting on dental service approachability. Appendix C: Figure S2: Variables corresponding to service-oriented model of accessing dental care. [file 12903_2021_1816_MOESM1_ESM.pdf]

Associations between dental care approachability and dental attendance among women pregnant with an Indigenous child: A cross-sectional study

Yuan Gao[1], Xiangqun Ju[2], Lisa Jamieson[2]*

Affiliation:

[1] School of public health, The University of Adelaide, South Australia 5000, Australia

Email address: yuan.gao02@student.adelaide.edu.au

Ph: +61 0433992751

[2] Australian Research Centre for Population Oral Health, AU

Email address: xiangqun.ju@adelaide.edu.au

*Correspondence:

Lisa Jamieson

Australian Research Centre for Population Oral Health, AU

The University of Adelaide, SA 5005

Australia Email addresslisa.jamieson@adelaide.edu.au

Ph: +61 8 8313 4611

Figure S1 A conceptual framework of access to health care by Levesque and colleagues [25]

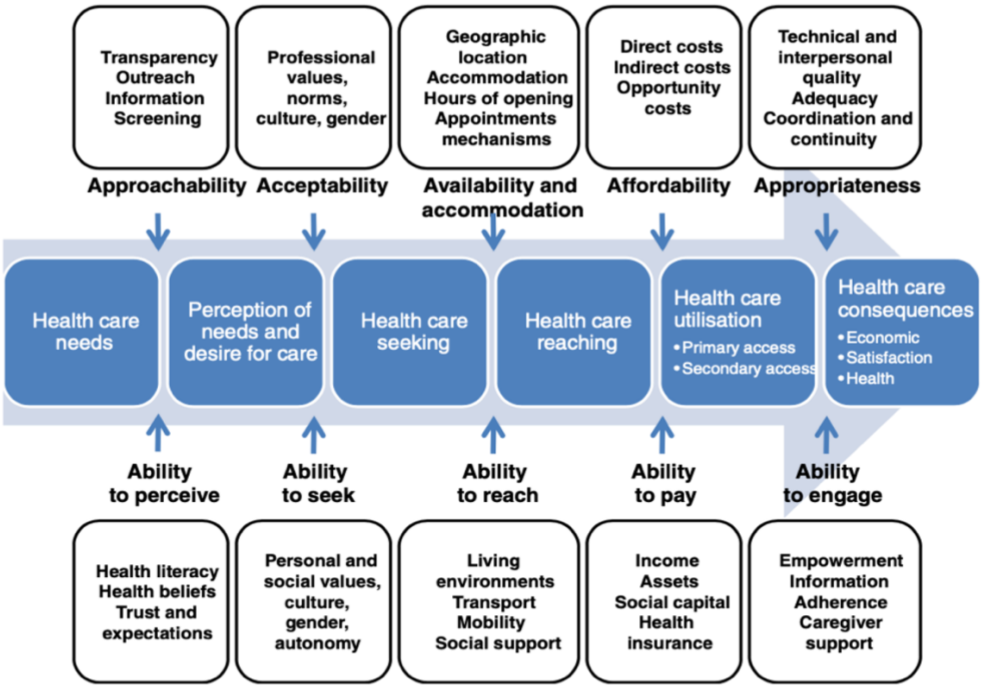

Figure S2 Variables corresponding to service-oriented model of accessing dental care

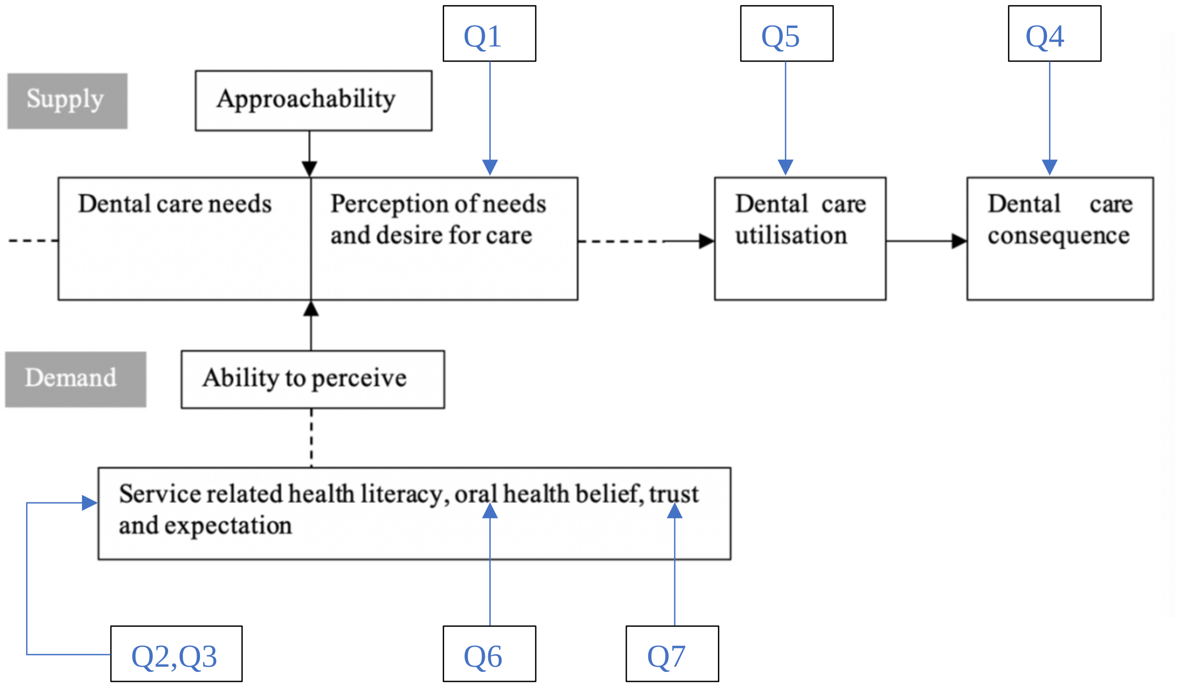

Table S1 Questionnaire of factors impacting on dental service approachability

	Questions/Answerers
	Perception of need and desire of care
Q1	Do you think you need to see a dentist?
	1. Yes<br>2. No
	Service-related health literacy<br>*(Oral health systems navigation: What to do when you want to see the dentist)*
Q2	If you needed to visit to the dentist tomorrow, would you know what to do?
	1. Yes<br>2. No
Q3	Do you think there would be a dentist able to see you tomorrow?
	1. Yes<br>2. No
	Dental care consequence
Q4	Do you think you have gum disease/bleeding gums?
	1. Yes<br>2. No
	Dental care utilisation
Q5	When did you last see a dentist?
	1. Less than one year ago<br>2. More than one year ago
	Oral health beliefs: How important do you rate the following in relation to teeth?
Q6	Visiting dentist
	1. Extremely important<br>2. Fairly important<br>3. Doesn't matter much<br>4. Not very important<br>5. Not at all important
	Trust and expectation to the service
Q7	I believe going to dentist would help my teeth.
	1. Strongly agree<br>2. Somewhat agree<br>3. Neither agree nor disagree<br>4. Somewhat disagree<br>5. Strongly disagree
